# 9β-Hy­droxy-1β,10α-ep­oxy­parthenolide

**DOI:** 10.1107/S1600536810033404

**Published:** 2010-08-25

**Authors:** Mohamed Moumou, Mohamed Akssira, Lahcen El Ammari, Ahmed Benharref, Moha Berraho

**Affiliations:** a Laboratoire de Chimie Bioorganique et Analytique, URAC 22, BP 146, FSTM, Université Hassan II, Mohammedia-Casablanca 20810 Mohammedia, Morocco; b Laboratoire de Chimie du Solide Appliquée, Faculté des Sciences, Avenue Ibn Battouta, BP 1014 Rabat, Morocco; c Laboratoire de Chimie des Substances Naturelles, URAC16. Faculté des Sciences Semlalia, BP 2390 Bd My Abdellah, 40000 Marrakech, Morocco

## Abstract

The title compound, C_15_H_20_O_5_ (systematic name: 5-hydroxy-1a,4a-dimethyl-7-methyleneperhydrodioxireno[5,6:9,10]cyclo­deca[1,2-*b*]furan-8-one), was obtained by the reaction of 3-chloro­perbenzoic acid with 9β-hy­droxy­parthenolide. The five-membered ring adopts a twist conformation, whereas the ten-membered ring displays an approximate chair–chair conformation. In the crystal structure, mol­ecules are linked into chains propagating along the *b* axis by inter­molecular O—H⋯O hydrogen bonds.

## Related literature

For background to the medicinal uses of the plant *Anvillea radiata*, see: Abdel Sattar *et al.* (1996 ▶); Bellakhdar (1997 ▶); El Hassany *et al.* (2004 ▶); Qureshi *et al.* (1990 ▶). For ring puckering parameters, see: Cremer & Pople (1975 ▶). For conformations of ten-membered rings, see: Castaneda-Acosta *et al.* (1997 ▶); Watson & Zabel (1982 ▶).
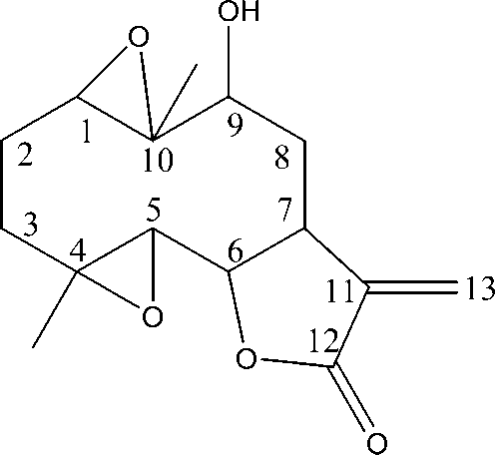


## Experimental

### 

#### Crystal data

C_15_H_20_O_5_
*M* *_r_* = 280.31
Monoclinic, 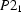

*a* = 9.2295 (3) Å
*b* = 9.5431 (3) Å
*c* = 9.3787 (3) Å
β = 118.662 (2)°
*V* = 724.84 (4) Å^3^
*Z* = 2
Mo *K*α radiation
μ = 0.10 mm^−1^
*T* = 298 K
0.27 × 0.18 × 0.12 mm

#### Data collection

Bruker X8 APEXII CCD area-detector diffractometer
8090 measured reflections
1617 independent reflections
1378 reflections with *I* > 2σ(*I*)
*R*_int_ = 0.031

#### Refinement

*R*[*F* ^2^ > 2σ(*F* ^2^)] = 0.033
*wR*(*F* ^2^) = 0.090
*S* = 1.05
1617 reflections
184 parameters
1 restraint
H-atom parameters constrained
Δρ_max_ = 0.13 e Å^−3^
Δρ_min_ = −0.13 e Å^−3^

### 

Data collection: *APEX2* (Bruker, 2005 ▶); cell refinement: *SAINT* (Bruker, 2005 ▶); data reduction: *SAINT*; program(s) used to solve structure: *SHELXS97* (Sheldrick, 2008 ▶); program(s) used to refine structure: *SHELXL97* (Sheldrick, 2008 ▶); molecular graphics: *ORTEP-3 for Windows* (Farrugia, 1997 ▶) and *PLATON* (Spek, 2009 ▶); software used to prepare material for publication: *WinGX* (Farrugia, 1999 ▶).

## Supplementary Material

Crystal structure: contains datablocks I, global. DOI: 10.1107/S1600536810033404/ci5157sup1.cif

Structure factors: contains datablocks I. DOI: 10.1107/S1600536810033404/ci5157Isup2.hkl

Additional supplementary materials:  crystallographic information; 3D view; checkCIF report

## Figures and Tables

**Table 1:** Hydrogen-bond geometry (Å, °)

*D*—H⋯*A*	*D*—H	H⋯*A*	*D*⋯*A*	*D*—H⋯*A*
O2—H2⋯O4^i^	0.82	2.02	2.787 (3)	155

Symmetry code: (i) 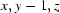.

**Fig. 1.**
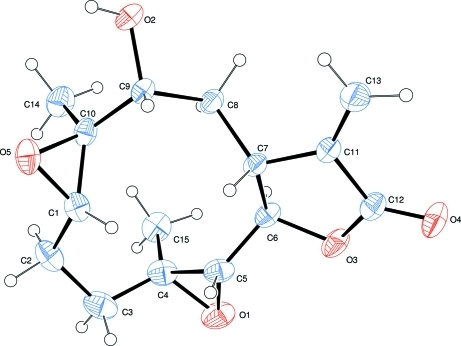
Molecular structure of the title compound, with the atom-labelling scheme. Displacement ellipsoids are drawn at the 30% probability level. H atoms are represented as small spheres of arbitrary radii.

**Fig. 2.**
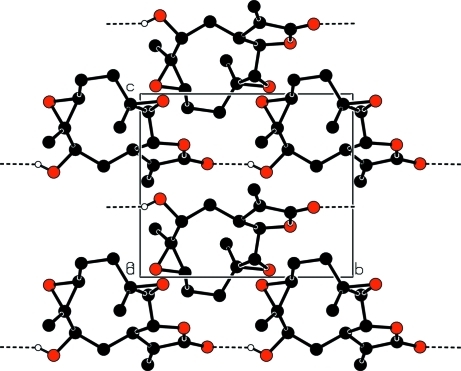
Partial packing view, showing C—H···O hydrogen-bonded chains parallel to the b axis. H atoms not involved in hydrogen bonding (dashed lines) have been omitted for clarity.

## supplementary crystallographic information

### Comment

*Anvillea radiata* Coss. & Dur. (Asteraceae) is a wild plant predominantly distributed in steppes of North Africa (Morocco and Algeria). The plant is used in the folk medicine for the treatment of dysentery, gastric-intestinal disorders (Bellakhdar, 1997), hypoglycemic activity (Qureshi *et al.*, 1990), and has been reported to have antitumoral activity (Abdel Sattar *et al.*, 1996). In our study of different Moroccan endemic plants, we have demonstrated that the aerial parts of *Anvillea radiata* Coss and Dur could be used as a renewable source of 9-hydroxyparthenolide (El Hassany, *et al.*, 2004). This work focuses on the preparation of 1β-10α-epoxy-9β-hydroxyparthenolide from the epoxydation of 9β-hydroxy parthenolide.

The molecular structure of the title compound is shown in Fig. 1. The five-membered ring adopts a twist conformation, as indicated by Cremer & Pople (1975) puckering parameters Q = 0.186 (3) Å and φ = 61.5 (8)°. The ten-membered ring displays an approximate chair-chair conformation. This is the typical conformation found in other sesquiterpenes lactones (Watson & Zabel, 1982; Castaneda-Acosta *et al.*, 1997).

In the crystal structure, molecules are linked into chains (Fig. 2) running along the *b* axis by intermolecular O—H···O hydrogen bonds (Table 1) involving the O2 and O4 atoms.

### Experimental

The title compound was obtained by treatment of 9β-Hydroxyparthenolide (500 mg) by *m*-chloroperbenzoic acid (250 mg) in CH_2_Cl_2_ (75 ml). The mixture was stirred for 30 min at room temperature and treated with aqueous solution of Na_2_CO_3_ (10%), then extracted by CH_2_Cl_2_. The residue obtained after evaporation of CH_2_Cl_2_, was chromatographed on a silica gel column with hexane-ethylacetate (50/50) as an eluent, to isolate 350 mg of the title compound in 75% yield. It was recrystallized from CH_2_Cl_2_ (m.p. 363–365 K). The structure of the compound was analyzed by ^1^H and ^13^C-NMR and confirmed by X-ray analysis.

### Refinement

All H atoms were positioned geometrically [C–H = 0.96 Å (methyl), 0.97 Å (methylene) and 0.98Å (methine)]and treated as riding with *U*_iso_(H) = 1.2U_eq_(methylene/methine C and O) or *U*_iso_(H) = 1.5U_eq_(methyl C). In the absence of significant anomalous scattering, the absolute configuration could not be reliably determined and thus 1278 Friedel pairs were merged.

### Crystal data

**Table:**

C_15_H_20_O_5_	*F*(000) = 300
*M**_r_* = 280.31	*D*_x_ = 1.284 Mg m^−^^3^
Monoclinic, *P*2_1_	Mo *K*α radiation, λ = 0.71073 Å
Hall symbol: P 2yb	Cell parameters from 8090 reflections
*a* = 9.2295 (3) Å	θ = 2.5–26.5°
*b* = 9.5431 (3) Å	µ = 0.10 mm^−^^1^
*c* = 9.3787 (3) Å	*T* = 298 K
β = 118.662 (2)°	Prism, colourless
*V* = 724.84 (4) Å^3^	0.27 × 0.18 × 0.12 mm
*Z* = 2	

### Data collection

**Table:**

Bruker X8 APEXII CCD area-detector diffractometer	1378 reflections with *I* > 2σ(*I*)
Radiation source: fine-focus sealed tube	*R*_int_ = 0.031
graphite	θ_max_ = 26.7°, θ_min_ = 2.5°
φ and ω scans	*h* = −11→11
8090 measured reflections	*k* = −10→12
1617 independent reflections	*l* = −11→11

### Refinement

**Table:**

Refinement on *F*^2^	Primary atom site location: structure-invariant direct methods
Least-squares matrix: full	Secondary atom site location: difference Fourier map
*R*[*F*^2^ > 2σ(*F*^2^)] = 0.033	Hydrogen site location: inferred from neighbouring sites
*wR*(*F*^2^) = 0.090	H-atom parameters constrained
*S* = 1.05	*w* = 1/[σ^2^(*F*_o_^2^) + (0.0541*P*)^2^ + 0.0327*P*] where *P* = (*F*_o_^2^ + 2*F*_c_^2^)/3
1617 reflections	(Δ/σ)_max_ = 0.001
184 parameters	Δρ_max_ = 0.13 e Å^−^^3^
1 restraint	Δρ_min_ = −0.13 e Å^−^^3^

### Fractional atomic coordinates and isotropic or equivalent isotropic displacement parameters (Å^2^)

**Table:**

	*x*	*y*	*z*	*U*_iso_*/*U*_eq_	
C1	−0.1569 (3)	0.2114 (3)	0.0240 (3)	0.0445 (5)	
H1	−0.0953	0.2863	0.0055	0.053*	
C2	−0.3412 (3)	0.2235 (3)	−0.0757 (3)	0.0567 (7)	
H2A	−0.3919	0.1709	−0.0227	0.068*	
H2B	−0.3757	0.1811	−0.1811	0.068*	
C3	−0.4043 (3)	0.3738 (4)	−0.1004 (3)	0.0638 (8)	
H3A	−0.3743	0.4211	−0.1741	0.077*	
H3B	−0.5238	0.3729	−0.1505	0.077*	
C4	−0.3354 (3)	0.4546 (3)	0.0564 (3)	0.0522 (6)	
C5	−0.1856 (3)	0.5389 (3)	0.0973 (3)	0.0493 (6)	
H5	−0.1470	0.5325	0.0167	0.059*	
C6	−0.0511 (2)	0.5623 (2)	0.2665 (3)	0.0421 (5)	
H6	−0.0915	0.5477	0.3448	0.051*	
C7	0.0972 (3)	0.4660 (2)	0.3050 (3)	0.0367 (5)	
H7	0.0907	0.4403	0.2009	0.044*	
C8	0.1049 (3)	0.3287 (2)	0.3942 (3)	0.0423 (5)	
H8A	0.2046	0.3294	0.4986	0.051*	
H8B	0.0116	0.3261	0.4149	0.051*	
C9	0.1036 (3)	0.1953 (2)	0.3041 (3)	0.0395 (5)	
H9	0.1641	0.2126	0.2439	0.047*	
C10	−0.0689 (3)	0.1455 (2)	0.1862 (3)	0.0407 (5)	
C11	0.2419 (3)	0.5625 (2)	0.3892 (3)	0.0399 (5)	
C12	0.1773 (3)	0.7075 (2)	0.3533 (3)	0.0465 (5)	
C13	0.3997 (3)	0.5360 (3)	0.4776 (3)	0.0601 (7)	
H13A	0.4749	0.6094	0.5198	0.072*	
H13B	0.4367	0.4438	0.4980	0.072*	
C14	−0.1584 (3)	0.0708 (3)	0.2633 (3)	0.0589 (7)	
H14A	−0.1411	0.1202	0.3593	0.088*	
H14B	−0.1164	−0.0229	0.2917	0.088*	
H14C	−0.2744	0.0675	0.1877	0.088*	
C15	−0.3799 (3)	0.4050 (3)	0.1816 (3)	0.0599 (7)	
H15A	−0.3367	0.4691	0.2715	0.090*	
H15B	−0.3336	0.3137	0.2190	0.090*	
H15C	−0.4979	0.4004	0.1347	0.090*	
O1	−0.3421 (2)	0.6072 (2)	0.0388 (3)	0.0650 (5)	
O2	0.1910 (2)	0.09367 (17)	0.4267 (2)	0.0569 (5)	
H2	0.1973	0.0204	0.3843	0.085*	
O3	0.0115 (2)	0.70565 (16)	0.2779 (2)	0.0539 (4)	
O4	0.2528 (2)	0.81591 (19)	0.3811 (2)	0.0653 (5)	
O5	−0.0845 (2)	0.07350 (19)	0.0434 (2)	0.0549 (4)	

### Atomic displacement parameters (Å^2^)

**Table:**

	*U*^11^	*U*^22^	*U*^33^	*U*^12^	*U*^13^	*U*^23^
C1	0.0426 (11)	0.0524 (14)	0.0442 (11)	−0.0061 (10)	0.0254 (9)	−0.0082 (11)
C2	0.0443 (13)	0.077 (2)	0.0458 (13)	−0.0123 (12)	0.0194 (10)	−0.0122 (13)
C3	0.0351 (12)	0.091 (2)	0.0544 (15)	0.0047 (12)	0.0123 (11)	0.0073 (14)
C4	0.0341 (11)	0.0619 (16)	0.0599 (15)	0.0111 (10)	0.0221 (10)	0.0078 (12)
C5	0.0391 (11)	0.0543 (14)	0.0561 (13)	0.0127 (10)	0.0241 (10)	0.0159 (11)
C6	0.0454 (11)	0.0330 (12)	0.0557 (13)	0.0072 (9)	0.0306 (10)	0.0073 (10)
C7	0.0431 (12)	0.0284 (10)	0.0398 (11)	0.0044 (8)	0.0210 (9)	0.0030 (8)
C8	0.0550 (12)	0.0300 (11)	0.0416 (11)	0.0007 (10)	0.0228 (9)	0.0020 (9)
C9	0.0446 (11)	0.0293 (10)	0.0465 (12)	0.0023 (9)	0.0232 (10)	0.0002 (9)
C10	0.0484 (12)	0.0347 (11)	0.0488 (12)	−0.0043 (9)	0.0311 (10)	−0.0063 (9)
C11	0.0450 (11)	0.0321 (11)	0.0442 (11)	0.0009 (9)	0.0227 (9)	0.0015 (9)
C12	0.0569 (13)	0.0331 (12)	0.0544 (13)	0.0020 (11)	0.0306 (11)	−0.0006 (11)
C13	0.0461 (13)	0.0519 (15)	0.0699 (16)	0.0010 (11)	0.0178 (11)	0.0071 (13)
C14	0.0654 (15)	0.0529 (16)	0.0695 (16)	−0.0123 (13)	0.0413 (13)	0.0045 (13)
C15	0.0536 (14)	0.0636 (17)	0.0754 (18)	0.0038 (13)	0.0413 (13)	0.0003 (13)
O1	0.0441 (10)	0.0637 (13)	0.0835 (12)	0.0202 (8)	0.0276 (9)	0.0214 (10)
O2	0.0673 (11)	0.0297 (8)	0.0630 (10)	0.0118 (8)	0.0226 (9)	0.0057 (8)
O3	0.0574 (10)	0.0299 (8)	0.0780 (11)	0.0121 (8)	0.0355 (9)	0.0071 (9)
O4	0.0786 (13)	0.0307 (8)	0.0896 (13)	−0.0083 (8)	0.0429 (10)	−0.0048 (9)
O5	0.0596 (9)	0.0517 (10)	0.0604 (10)	−0.0073 (8)	0.0342 (8)	−0.0212 (8)

### Geometric parameters (Å, °)

**Table:**

C1—O5	1.447 (3)	C8—C9	1.525 (3)
C1—C10	1.477 (3)	C8—H8A	0.97
C1—C2	1.502 (3)	C8—H8B	0.97
C1—H1	0.98	C9—O2	1.422 (3)
C2—C3	1.524 (4)	C9—C10	1.515 (3)
C2—H2A	0.97	C9—H9	0.98
C2—H2B	0.97	C10—O5	1.449 (3)
C3—C4	1.505 (4)	C10—C14	1.512 (3)
C3—H3A	0.97	C11—C13	1.310 (3)
C3—H3B	0.97	C11—C12	1.480 (3)
C4—O1	1.463 (3)	C12—O4	1.204 (3)
C4—C5	1.482 (4)	C12—O3	1.343 (3)
C4—C15	1.495 (4)	C13—H13A	0.93
C5—O1	1.432 (3)	C13—H13B	0.93
C5—C6	1.490 (3)	C14—H14A	0.96
C5—H5	0.98	C14—H14B	0.96
C6—O3	1.469 (3)	C14—H14C	0.96
C6—C7	1.541 (3)	C15—H15A	0.96
C6—H6	0.98	C15—H15B	0.96
C7—C11	1.498 (3)	C15—H15C	0.96
C7—C8	1.537 (3)	O2—H2	0.82
C7—H7	0.98		
			
O5—C1—C10	59.41 (15)	C9—C8—H8A	108.5
O5—C1—C2	117.7 (2)	C7—C8—H8A	108.5
C10—C1—C2	124.9 (2)	C9—C8—H8B	108.5
O5—C1—H1	114.4	C7—C8—H8B	108.5
C10—C1—H1	114.4	H8A—C8—H8B	107.5
C2—C1—H1	114.4	O2—C9—C10	111.51 (18)
C1—C2—C3	113.8 (2)	O2—C9—C8	105.69 (16)
C1—C2—H2A	108.8	C10—C9—C8	113.10 (17)
C3—C2—H2A	108.8	O2—C9—H9	108.8
C1—C2—H2B	108.8	C10—C9—H9	108.8
C3—C2—H2B	108.8	C8—C9—H9	108.8
H2A—C2—H2B	107.7	O5—C10—C1	59.26 (15)
C4—C3—C2	112.5 (2)	O5—C10—C14	112.5 (2)
C4—C3—H3A	109.1	C1—C10—C14	122.4 (2)
C2—C3—H3A	109.1	O5—C10—C9	115.25 (17)
C4—C3—H3B	109.1	C1—C10—C9	119.00 (18)
C2—C3—H3B	109.1	C14—C10—C9	115.0 (2)
H3A—C3—H3B	107.8	C13—C11—C12	121.9 (2)
O1—C4—C5	58.19 (16)	C13—C11—C7	130.9 (2)
O1—C4—C15	113.1 (2)	C12—C11—C7	107.19 (18)
C5—C4—C15	122.8 (2)	O4—C12—O3	121.4 (2)
O1—C4—C3	115.1 (2)	O4—C12—C11	128.7 (2)
C5—C4—C3	115.8 (2)	O3—C12—C11	109.87 (19)
C15—C4—C3	117.3 (2)	C11—C13—H13A	120.0
O1—C5—C4	60.23 (15)	C11—C13—H13B	120.0
O1—C5—C6	120.8 (2)	H13A—C13—H13B	120.0
C4—C5—C6	123.7 (2)	C10—C14—H14A	109.5
O1—C5—H5	113.9	C10—C14—H14B	109.5
C4—C5—H5	113.9	H14A—C14—H14B	109.5
C6—C5—H5	113.9	C10—C14—H14C	109.5
O3—C6—C5	108.32 (18)	H14A—C14—H14C	109.5
O3—C6—C7	105.30 (16)	H14B—C14—H14C	109.5
C5—C6—C7	110.70 (18)	C4—C15—H15A	109.5
O3—C6—H6	110.8	C4—C15—H15B	109.5
C5—C6—H6	110.8	H15A—C15—H15B	109.5
C7—C6—H6	110.8	C4—C15—H15C	109.5
C11—C7—C8	116.15 (18)	H15A—C15—H15C	109.5
C11—C7—C6	102.94 (17)	H15B—C15—H15C	109.5
C8—C7—C6	116.07 (17)	C5—O1—C4	61.57 (16)
C11—C7—H7	107.0	C9—O2—H2	109.5
C8—C7—H7	107.0	C12—O3—C6	111.09 (16)
C6—C7—H7	107.0	C1—O5—C10	61.33 (14)
C9—C8—C7	115.12 (17)		

### Hydrogen-bond geometry (Å, °)

**Table:**

*D*—H···*A*	*D*—H	H···*A*	*D*···*A*	*D*—H···*A*
O2—H2···O4^i^	0.82	2.02	2.787 (3)	155

Symmetry codes: (i) *x*, *y*−1, *z*.
